# Perspectives of parents on the meaning of happiness in children with long‐term illness: A hybrid concept analysis

**DOI:** 10.1002/nop2.532

**Published:** 2020-06-17

**Authors:** Ruth Nimota Nukpezah, Mohammad Ali Cheraghi, Shahzad Pashaeypoor, Akram Sadat Sadat Hoseini

**Affiliations:** ^1^ Department of Pediatric Nursing, School of Nursing & Midwifery International Campus ‐ Tehran University of Medical Sciences, Iran Tehran Iran; ^2^ Research Center for Quran, Hadith, and Medicine, Spiritual Health Group Tehran University of Medical Sciences Tehran Iran; ^3^ Department of Community Health and Geriatric Nursing School of Nursing and Midwifery Tehran University of Medical Sciences Tehran Iran; ^4^ Department of Pediatric Nursing School of Nursing and Midwifery Tehran University of Medical Sciences Tehran Iran

**Keywords:** children, chronic disease, concept analysis, happiness, hybrid approach, parent's perspectives

## Abstract

**Aim:**

To define the concept of happiness among children with chronic disease.

**Background:**

Happiness is an old human quest, the existing literature on the definition of happiness among children with chronic diseases is sparse.

**Methods:**

The three‐phase hybrid model was used. In the first (theoretical) phase, a literature review was conducted. In the second (fieldwork) phase, the semi‐structured interview data were analysed through content analysis. Ten participants were purposively sampled. In the third (final analytic) phase, the practical definition of the concept was identified.

**Results:**

The practical definition of happiness in a Ghanaian chronically ill child is the “subjective and positive lifelong process of adapting to biological, physiological, psychosocial, economic and environmental changes caused by the disease trajectory, which affects the well‐being of the child and the family.”

**Conclusions:**

This study offers a background for selecting appropriate health indicators and outcome measures in promoting happiness in children with chronic diseases.

## 1 INTRODUCTION

Over the past century, the prevalence of long‐term illnesses/chronic diseases among children and young people has increased relative to infectious diseases. This situation has left the children with such long‐term illness and their families unhappy (Lim, 2019; Ray, 2002; Thorne & Paterson, 1998; Wijlaars, Gilbert, & Hardelid, 2016). A long‐term illness is a sickness that lasts more than three months (Hockenberry & Wilson, 2015); it affects a person's normal activities, often requires hospitalization and results in extensive medical care needs (Wijlaars et al., 2016). Long‐term illnesses, such as asthma, obesity, mental health conditions, leukaemia, sickle cell disease and respiratory conditions, have been seen to threaten our normative ideas of happiness (e.g. life satisfaction, felicity, health, well‐being and comfort) (McCormick & Cushman, 2019). Children with chronic diseases are not happy and satisfied with their lives, when compared to their healthy peers (Post, 2005). In addition to this, chronic diseases in children cause delays in developmental milestones in areas such as physical, language and communication, reasoning, social and emotional growth (Lim, 2019). These delays affect the child, the family members and the nation as a whole due to the resulting economic and social burdens and personal feeling of gross unhappiness (Lim, 2019). So what are the definition and the attributes of happiness in children facing a tenacious ailment like chronic disease? Happiness has been defined as the degree to which an individual judges the overall quality of their own life (Ozkara, 2015; Rinnan et al., 2018). Meanwhile, other researchers also believe that happiness is a two‐dimensional construct involving both hedonic (positive emotions and general satisfaction with life) proposed by Carruthers & Hood (2004) and Cantor, Sanderson, Kahneman, Diener, & Schwartz (1999) and a eudaimonic perspective (a belief that happiness is the fulfilment of positive psychological functioning and human development) (Seligman, 2011). Seligman (Peterson, Park, & Seligman, 2005) also stated that happiness is cumulative and it can exist even when we are not comfortable, fit or satisfied with life. The concept of nurses’ happiness (NH) has been well described (Meng, Luo, Liu, Hu, & Yu, 2015; Ozkara, 2015). However, there is no complete understanding of the meaning of happiness among children with chronic diseases (Ngamaba, Panagioti, & Armitage, 2017). Furthermore, each of the previous studies in the area of happiness revealed only some aspects of the concept of the happiness (Meng et al., 2015; Oishi, Graham, Kesebir, & Galinha, 2013; Ozkara, 2015; Post, 2005), and hence, there is no complete understanding about the different aspects of the concept such as what happiness means in children with chronic diseases. Moreover, the attributes of the concept of happiness among children with long‐term illness greatly depend on: the immediate sociocultural context, the qualities of the immediate healthcare system and the type of chronic ailment the children and their families are battling with. All of these aforementioned factors influenced the decision to further classify the concept of happiness among the children living with chronic health conditions in Ghana. In addition, anecdotal evidence has suggested that exploring the different aspects of the concept of happiness in children with chronic conditions and providing a clear definition for happiness based on the immediate context are the prerequisites for the effective fulfilment of happiness (Thorne & Paterson, 1998). Indeed, given the efforts made in various disciplines to clarify the concept of happiness (Ray, 2002; Thorne & Paterson, 1998; Veenhoven, 2008), it can be inferred that the necessity and importance of happiness as the essence of nursing care are well acknowledged. A study on achieving happiness in chronic health diseases by McCormick (McCormick & Cushman, 2019) attributed the emotion (happiness) of children with a long‐term illness to “positive health assets,” McCormick defined happiness as a process of strengthening the abilities of a child with chronic disease to adjust to environmental challenges, gratify needs and attain goals to improve the well‐being of the child and the family (McCormick & Cushman, 2019). Nonetheless, the attribute and the definition of this concept remain controversial and there are lots of inconsistencies in its definition; hence, it is deemed worthy of attention in nursing practice (Ferrer‐Cascales et al., 2019; Ozkara, 2015; Rinnan et al., 2018), especially in children with chronic diseases. Additionally, self‐reporting of happiness is problematic for adults and even more so for a young child who is less skilled in placing instant feelings into a longer perspective. The parents of children with chronic diseases are better positioned to describe what happiness means to their children (Ray, 2002), caregivers are the appropriate guide to fall on in defining the meaning of happiness in their children (McCormick & Cushman, 2019). Thus, this paper explored the definition of happiness in children with chronic diseases using a hybrid concept analysis research method.

### 1.1 Objectives

To define the concept of happiness in children with chronic diseases so as to attain an in‐depth understanding about the concept in the sociocultural context of Ghana.

## 2 METHOD

### 2.1 Study design

In this study, the concept of happiness among children with chronic disease was analysed using Schwartz‐Barcott and Kim's hybrid model. This model of concept analysis consists of three phases: theoretical phase, fieldwork phase and final analysis phase (Schwartz‐Barcott & Suzie Kim, 2000). We chose the hybrid concept analysis method because its qualitative inclusion of parents’ perspectives about what happiness means to their children enriched the inadequate research literature in this field.

### 2.2 The theoretical analysis of the concept of happiness

Theoretical analysis is the first phase in an hybrid concept analysis (Schwartz‐Barcott & Suzie Kim, 2000). The aim of this phase is to deepen the understanding of the concept of happiness in children with chronic diseases based on the existing works of research. The four steps in theoretical analysis described by Schwartz‐Barcott and Kim (Schwartz‐Barcott & Suzie Kim, 2000), namely selecting a concept, searching the literature, looking for meaning and measurements and finally coming out with a working definition, were used in this current study (Schwartz‐Barcott & Suzie Kim, 2000).

#### 2.2.1 Data collection procedure

A literature review and a face‐face interviews were conducted (Schwartz‐Barcott & Suzie Kim, 2000).

##### Search strategy

A literature review was conducted using the peer‐reviewed English language journal databases of CINAHL, ProQuest, Web of Science, Scopus, PubMed and Google Scholar. In addition, the literature search was not limited by start date and all literature up to December 2019 was included. The keywords of “happiness/unhappiness,” “health,” “well‐being,” “chronic illness,” “parents’ perspective” and “children” were included in the search of the articles.

##### Inclusion criteria

Studies written in English language on the definitions of happiness in chronically ill children were included during the search.

Exclusion criterion: Studies published in non‐English language were excluded.

##### Article types

The articles published on the subject of happiness and associated emotions such as well‐being and health among children with chronic diseases in the areas of quantitative, qualitative, meta‐analysis, books, meta‐synthesis and exemplar studies were searched.

#### 2.2.2 The review of the studies was based on the following questions

How has happiness been defined? And how has happiness among the chronically ill child been conceptualized and measured? Each document relieved was read word by word, line by line and paragraph by paragraph for several times to obtain a general understanding about it using the method of content analysis (Kyngäs, 2020). Trustworthiness of the data was ensured through explicitly expressing the study aim, explaining the search strategy and examining the appropriateness of the retrieved documents (Kyngäs, 2020; Kyngäs, Kääriäinen, & Elo, 2020). The analysis of the literature reviewed in this phase ended with a working definition of the concept of happiness among children with chronic diseases from the theoretical perspectives.

### 2.3 The fieldwork phase

This is the second phase of the hybrid concept analysis (Schwartz‐Barcott & Suzie Kim, 2000). It helped to further explore and empirically validate the theoretical findings of the definition of the concept of happiness among the chronically ill children in Ghana by analytical development, which had begun at the theoretical phase. The aim of this phase is to strengthen and modify the concept by the use of a qualitative research method and with a content analysis approach (Kyngäs, 2020; Kyngäs et al., 2020).

#### 2.3.1 Data collection procedure

A descriptive qualitative study was conducted to gather information from the participants. This stage included the fundamental steps that are common in qualitative research conducted by Schwartz‐Barcott and Kim (Kyngäs, 2020; Schwartz‐Barcott & Suzie Kim, 2000). The stage involved three steps: (a) preparing the conditions; (b) selecting the samples; and (c) collecting and analysing the data (Schwartz‐Barcott & Suzie Kim, 2000).

##### Preparing the conditions

In preparing the conditions, the emphasis of this step was on selecting the field setting and identifying the main questions.

##### Fieldwork settings

The field setting for this study was the children's ward of Tamale Teaching Hospital in Ghana. This hospital is a referral centre; hence, its paediatric ward had variety of cases, some of which presented the chronic conditions that enriched the study.

##### Fieldwork questions

The primary questions were the same as in the theoretical phase, and further questions were generated from the participants’ responses. The questions that were identified for interviewing the parents of children with chronic diseases were as follows:

1. “What is your understanding of the meaning of happiness in relation to your child's condition?”

After the first responses, explorative and reflective statements were used to follow the participants’ explanation of their perspectives of the concept of happiness such as: What does it mean for your child to be happy? What kind of feeling do you have regarding whether your child is happy? What kind of effect has your child’s ill‐health state had on his level of being happy? And what are the facilitators that help to promote your child’s rate of being happy?

##### Participants and sample size selection

The participants were parents of children with chronic diseases. The parents were the appropriate participants for this study because self‐reporting of happiness is problematic for young children, because they are less skilled in placing instant feelings into a longer perspective. Parents of children with chronic ailments are therefore better positioned to describe what happiness means to their children (Ray, 2002). Participants serve to provide a thorough reflection of a chosen concept (Kyngäs, 2020; Schwartz‐Barcott & Suzie Kim, 2000). Thus, ten (Carruthers & Hood, 2004) parents of school‐aged children with diverse chronic ailments were purposively selected and interviewed. The children were between the ages of 6–11 years. The data were collected in 6 months, beginning from July–December 2019.

##### Data collection method

A face‐to‐face, in‐depth and semi‐structured interview was used to collect data from parents of children with chronically ill conditions. The inclusion criteria for the study were as follows: (a) being a parent of a chronically ill child; (b) children being admitted at the Tamale Teaching Hospital, Ghana; and (c) parents willingness to participate and give their provision of written and oral consent.

Interviews lasted for 30–35 min on average and were recorded. Data saturation was established in the last three interviewee's additional interviews, and my observations did not add new information. Several processes were taken to ensure trustworthiness using the Guba and Lincoln (1986) standards for ensuring rigour such as credibility, transferability, transparency and conformability (Diener, Emmons, Larsen, & Griffin, 1985), only one researcher after collecting the interview transcribed it. Two participants dropped out during the interview section due to the severity of the children's conditions; thus, 80% participated to the end.

##### Field data analysis

Data collection and analysis were carried out simultaneously. The qualitative content analysis was used for this study (Kyngäs, 2020). This analysis method was deemed important because it is useful for interpreting the content of textual data (Kyngäs, 2020; Kyngäs et al., 2020).

### 2.4 The final analytic phase

In the analytic phase, findings from the fieldwork phase were compared with the theoretical phase data to produce a refined definition of the concept of happiness among children with chronic health condition supported by both the literature and the participant's perspectives. Afterwards, the common meanings of the concept of happiness in children with chronic diseases were identified and reported as the operational definition of the concept.

### 2.5 Ethical consideration

The ethics for this study was granted by the Tamale Teaching Hospital (TTH) in Ghana, Ref: TTH/R&D/SR/125. After explaining the purpose of the study, written and verbal informed consent was obtained from all participants in accordance with the code of ethics of the Helsinki Declaration.

## 3 RESULTS

### 3.1 Definition of happiness in children with chronic diseases from a theoretical perspective

From the concise Oxford dictionary (Allen, Fowler, Fowler, & McIntosh, 1990), happiness is defined as a state of well‐being and fulfilment of a enjoyable and satisfying feeling, with no objection to something. Numerous words were found in a thesaurus to describe the concept of happiness such as enjoyment, felicity, excitement, optimism, gladness, contentment and positive well‐being. Domocmat (Lyubomirsky, King, & Diener, 2005) believed that happiness is related to the awareness of a transcendent being, an idea that one's life is good and worthwhile with a clearly defined meaningful purpose based on the cultural context of the person (Peterson et al., 2005), while Thorne & Paterson (1998) saw happiness to be courage and hope; the reshaping of the self; the regaining of control; the discovery of meaning; the empowerment of potential; and normality. Happiness in children with chronic diseases means the children and families counting and appreciating meaningful achievements made despite their current pressing ailment, dealing with the misfortune of sickness, fostering idealist reflection, enjoying the little progress made through the journey of the ailment (Mackner, Sisson, & Crandall, 2004; McCormick & Cushman, 2019), forming relationships, binding to significant life goals, active participation in one's religious rituals and engaging in physical activity (Lyubomirsky et al., 2005; Mackner et al., 2004). Firstly, a study on achieving happiness in chronic health by McCormick opined that children can discover a internal happiness even when they are down with long‐term illness (McCormick & Cushman, 2019). The study further stated that there are five multidimensions to happiness in children with chronic diseases. These include aspects of: positive emotion, engagement, meaning, accomplishment and relationship (McCormick & Cushman, 2019; Seligman, 2011). Secondly, according to McCormick (McCormick & Cushman, 2019), in practising positive emotions, the chronically ill child begins to accept their disease state and sets realistic targets for internal achievements (e.g. fulfilment). Children with chronic diseases practise engagement through an innate motivation for change, and they find substitutes to replace previous enjoyable activities. Thus, they become engrossed in a new set of activities that they become unaware that so much time has passed (McCormick & Cushman, 2019). Thirdly, Mccormick mentioned that happiness means building a good relationship. Hence, when children with chronic diseases get good and stable interpersonal relationship from families and peers, it helps in consoling them in time of pain. They often get supported by the words of encouragement they get from the identified support persons. Through this interpersonal relationship, a child will get the opportunity to freely talk about their illness with the support persons (caregivers and friends). This type of relationship helps to decrease their present worries, and it acts as a drive for living happily (McCormick & Cushman, 2019; Peterson et al., 2005). Fourthly, McCormick stated that finding “meaning” is the ability of the individual to judge what the disease means to them and this meaning can take numerous forms over the course of time. Nevertheless, caregivers can serve as a useful exemplar for a child during this period. “Meaning” is best measured by events that the child often engage in at most times (McCormick & Cushman, 2019). Long‐term illnesses often take a toll on the child and the child's family quality of life. Thus, the child is required to personally consider what is truly valued and meaningful to him/her (McCormick & Cushman, 2019), and which activities are more important. Hence, there is a need to build counts of meaningful achievements of their daily life (McCormick & Cushman, 2019; Peterson et al., 2005). Lastly, McCormick stated that happiness means the feeling of accomplishments. Therefore, parents can help expose the child to activities that they love and can easily master, for example engaging them in spellings and solving simple mathematical questions based on their ability (McCormick & Cushman, 2019). Regular reminders of this mastery help the child to have a sense of accomplishment (Allen et al., 1990; Mackner et al., 2004).

From the analysis of the works of literature, it was seen that participants construct a paradigm that they consider appropriate for their children and relate the type and severity of their children's ailment to such paradigm. Thus, happiness among children with chronic diseases is a personal evaluation rather than an evaluation based on some externally standardized objective criteria. After analysing the literature review on the definitions of the concept of happiness among children with chronic diseases, three critical attributes were grasped. Additionally, the antecedents, consequences and the empirical measurement of the concept of happiness among children with chronic diseases were also recognized.

#### 3.1.1 The attributes of the concept of happiness in children with chronic diseases

Attributes are defined as critical features (signs and symptoms) of a concept, which appears again and yet again during the analysis of the concept. It helps to distinguish one concept from another interrelated concept and clarify its meaning (McCormick & Cushman, 2019). There were three attributes identified from the works of literature. Firstly, most researchers agree that happiness in children with chronic diseases is a “subjective assessment of capabilities” (Mackner et al., 2004; McCormick & Cushman, 2019; Peterson et al., 2005; Post, 2005; Veenhoven, 2008). This attribute is considered vital for happiness in children with chronic diseases. Secondly, some researchers have mentioned happiness in children with chronic diseases as the act of “nurturing social prosperity against the odds” (Lyubomirsky et al., 2005; Mackner et al., 2004; McCormick & Cushman, 2019; Meng et al., 2015). Lastly, other researchers have attributed happiness in children with chronic diseases to “hopefulness” (Carver & Scheier, 2014; Cella & Nowinski, 2002; Eiser & Morse, 2001; Lyubomirsky et al., 2005; McCormick & Cushman, 2019; Meng et al., 2015; Scheier, Weintraub, & Carver, 1986).

#### 3.1.2 Antecedents

Antecedents are actions or instances that must occur first hand before a concept can result. According to Veenhoven (Veenhoven, 2008), the antecedents affecting the occurrence of happiness in a paediatric chronically ill child depend on the child's developmental stage, personality, coping mechanisms, the chronic disease condition itself (the type and the stage of the condition) and the family/friend/peer conducts and behaviour towards the chronically ill child. Other antecedents identified were as follows: social background of the child such as sex, age and socio‐economic status; type of personality such as extraversion, neuroticism and openness; and social network such as intimate attachments and friendship network (Headey & Wearing, 1992).

#### 3.1.3 Consequences of long‐term illness on a child's happiness/unhappiness state

The consequence of long‐term illness in a child is wide‐ranging depending on the chronicity of the condition. The family of a child with a chronic illness is often presented with additional tasks, responsibility and concerns (Ray, 2002). The child might experience difficulty in: communication, mobility, self‐care, temperament, communicating and socializing with friends, emotional well‐being and gaining independence/future aspirations. Most children lag in reaching various developmental milestones. The child's school and routine extracurricular activity at school can be affected. The child might be at risk for behaviour or emotional problems, and parents might be emotionally, socially, physically and financially handicapped as they struggle to cope with the care of the child. Siblings might also feel anger/jealous or guilty towards their sick sibling. Thus, happiness in the chronically ill child is often seen as the child and the families are able to adapt to the child's ill‐health condition. In view of children who are unhappy, the consequence could be difficulty in coping with their changing health, lack of adaptation and satisfaction with life (Singer, 1999). Persistent unhappiness leads to harmful effects, such as elevated blood pressure and low immune response. There are also signs that a positive mentality that is often seen in children who are happy can help prevent disease occurrence; for example, when one's mood is good, the immune response is often positive as well (Lyubomirsky et al., 2005). Another common mechanism is better health behaviour. Happy people are more likely to observe their weight (Mackner et al., 2004), feel more keenly about the symptoms of the disease (Carver & Scheier, 2014) and better respond to threatening information (Scheier et al., 1986). Happy people are also healthier, and they are more often engaged in physical activity (Mackner et al., 2004). Happier people also engage in health promotion behaviours. The opposite is true in people with depression, who tend to function slowly, possibly because they are more likely to get sick (Cella & Nowinski, 2002).

#### 3.1.4 Measurement of the concept

Measurement of the concept in the theoretical concept analysis phase aims to find empirical references for the attributes identified (Walker & Avant, 2011). According to Nodding (Noddings, 2003), happiness in children can be evaluated using a subjective and objective standard. Meanwhile, there is a lack of standardization in the conceptualization and operationalization of the concept of happiness in children and instruments developed for adults are less likely to be suitable for use in children. The health‐related quality of life (HRQOL) tool could be used as an assessment tool in children with chronic diseases. Some of the reference tools seen in the works of literature reviewed are the PedsQL™ (Pediatric Quality of Life Inventory™), Child Health Questionnaire (CHQ), and Child Health and Illness Profile (CHIP). Other instruments include the following: Personal Well‐Being Index—Adult (the PWI‐A), used to measure hedonic life satisfaction; and the Basic Needs Satisfaction in General Scale (BNSG‐S), used to evaluate eudaimonic well‐being (Johnston & Finney, 2010). According to Kozma and Stones (Kozma & Stones, 1980), Memorial University of Newfoundland Scale of Happiness (MUNSH) is also used to describe eudaimonic PWB and mental health. Other scales include Ways of Life Questionnaire (Peterson et al., 2005), Aspiration Index (Kasser & Ryan, 1996), the Satisfaction With Life Scale (Diener et al., 1985) and the Scales of Psychological Well‐Being (Ryff, 1989).

#### 3.1.5 Working definition

Based on the review of the literature, a working definition of the concept of happiness in children with chronic diseases was constructed as a “global assessment seen in the maintenance of balance in developmental self‐autonomy, engagement, meaning, accomplishment and the relationship used in coping with a chronic disease trajectory that affects the physiological, psychological and social well‐being of a child and his/her family.”

### 3.2 Results from the fieldwork phase

The attributes of the concept of happiness that were extracted from the fieldwork data were: “Positive quality of life,” “Subjective well‐being,” “Dependence on God,” “Physiological and economic stability” and “Optimism.”

The details of the attributes of happiness derived from participant's narratives are as follows:

#### 3.2.1 Positive quality of life

The findings show that some participants deem happiness as a state of healthy and sound mind and they also see health as a continuum between positive and negative poles and to be healthy means their child should be at the positive pole:

…There is no drop of happiness in me or my child. Happiness is when your mind is free, everything is going on normal, your child does not have health problems. You have sound mind if nothing is bothering you such as chronic conditions affecting any of my children and my child is able to breathe well without any difficulty… (a mother of a 11‐year‐old male child, with a diagnosis of chronic heart disease and bronchopneumonia).

#### 3.2.2 Subjective well‐being

Some parents consider happiness as a subjective concept and thus a personal explanation of the current state of their children's health. This emphasizes the basic assertion that personal differences could influence an individual choice of what is important or what they find important. Some people are satisfied with the smallest signs of recovery, while other people expect the best from everything, such as full recovery from the ill health of their children:

…The way she is laying down on the bed, I can say that she is not happy, unlike before her ill health, she could get up and run around. Although her eating habit has improved, I will be much happier if she could eat more and run around the way she used to do before the sickness. Since morning, she has taken only tea… (a mother of a 10‐year‐old female child, with the diagnosis of a nephrotic syndrome).

#### 3.2.3 Dependence on God

The participant believed that happiness is not controlled by humans. And it is only the supreme being that makes you happy:

…..I have left everything to God, Happiness is when somebody is not crying or worried about anything, I and my child get food and eat to our capacity. I think my child is happy because children cannot think of anything so, they cannot explain whether they are happy or not. … (a mother of a 6‐year‐old male baby, with a diagnosis of Septic Arthritis of the knee).

….Happiness is when the family accepts the child's condition without any reproach and accepts that God is in control despite the present chronic and debilitating illness…. (a mother of a 6‐year‐old male child, with the diagnosis of asthma).

…My child's condition has subsided now. I think to be happy; you have to encourage yourself and believe that God will definitely heal you. My child is now happy because he laughs so loud and plays with his siblings, this is the condition that defines happiness for me … (a father of an 8‐year‐old male baby, with the diagnosis of chronic airway obstruction).

#### 3.2.4 Physiological and economic stability

Some participants opined that happiness comprises physiological and economic aspects. Being healthy constitutes the physiological phase of happiness, while having wealth was defined as the economic phase because they believed that without wealth, they will not be able to afford treatment for the social health needs of life, including buying medications and other allied interventions that will be needed for the child:

….I and my child are happy because I am able to buy medications and anything that my child needs to support him to breathe. I am able to hold him upright in other to assist him to breathe. I am not happy because am not able to do my business; my child cannot breathe well except with support; my child is not able to go to school. In defining happiness, there are two things involved, healthiness or wealth to provide your need to cure for the illness. You are unhappy when you are worried about something and if you cannot provide for yourself. I am happy because now my child is breathing well, at first, he was finding it difficult to breathe. When you are unhappy you will be having negative thoughts and you will not be able to provide the needs to meet what the condition is demanding. I don't think my boy is happy, because of his intermittent breathing problem…… (a father of a 7‐year‐old male baby, with the diagnosis of chronic airway obstruction).

….Happiness is when you are healthy, you have money. Each day you can provide for yourself, you are not worried about anything. It will bring you happiness if you can provide for yourself and the sick person… (a mother of a 14‐year‐old boy, with the diagnosis of meningoencephalitis).

#### 3.2.5 Optimism

The value of hopefulness and expectation also featured in some of the family's assertions of what happiness means to them. These values helped these families to be able to endure the stress of nursing a child having long‐term illness. These families believed that showing signs of positive thinking, expectation and anticipation of recovery of their children would help cure the chronic condition:

….The way my child is laying down, how can she be happy? I am not also happy because this is not my hometown and am a stranger. If I need something whom do I go to? My child's condition is also affecting her school because of the condition she has dropped out of school. For me, happiness is the anticipation that everything will get better and I often dream and say to myself that this sickness will go away and will soon be a thing of the past. … (a mother of a 10‐year‐old female child, with the diagnosis of a nephrotic syndrome).

…Happiness leads a person towards hope, development and anticipation of a positive life. This concept aids me to nurture the drive and strength to endure and come to terms with the restrictions related to my Childs's chronic ailment….. (a mother of a 11‐year‐old boy, with the diagnosis of chronic heart disease and bronchopneumonia).

#### 3.2.6 Summaries of the antecedents and consequences of happiness from the fieldwork

This section highlights the summaries of the factors that precede happiness (antecedents) that were extracted from the fieldwork and the consequences of happiness/unhappiness in children with chronic diseases. The detailed write‐up for this section on the antecedents and consequences of happiness in children with chronic diseases will be further explained in another related article. The antecedents from most participants in the fieldwork analysis were as follows: “personal strengths of the child and family,” “type and stage of ailment,” “motivations” and the environmental conditions (“social–structural variables” and “cultural ones”), while the consequences of unhappiness from the field study were as follows: “feeling of guilt, hurt, sad, apprehension,” “low self‐confidence,” “loss of role‐function,” “questions about meaning in life and religious struggles.” In the same vein, the consequences of happiness resulted in the feeling of positive well‐being of the child, hopefulness, resilience and inner strength and ability to cope with the associated symptoms of the long‐term illness in the child, the parents believed that these consequences can lead to a child's fast recovery.

## 4 FINAL ANALYTIC PHASE

This is the third major and final stage of the hybrid concept analysis. In this phase, results from the fieldwork phase were harmonized with the result from the theoretical phase so as to bring out an advanced practical definition of happiness as opined by both the findings from the literature and the parents’ perspectives on children with chronic health conditions so as to come out with a practical definition of happiness among children with chronic diseases (Schwartz‐Barcott & Kim) (Schwartz‐Barcott & Suzie Kim, 2000). An analytic induction technique was deemed appropriate to analyse the qualitative report findings. This analysis brings about new empirical findings to be constantly matched and juxtaposed with the initial working definition of the concept of happiness. These new data are then used to validate and revise the theoretical ideas embedded in the current working definition and thus lead to the practical definition of the concept (Cantor et al., 1999).

### 4.1 Practical definition

Happiness in Ghanaian chronically ill child from the parent's perspective “is the subjective and positive lifelong process of adapting to bio‐psychosocial, physiological, economic and environmental changes caused by the disease trajectory, which affects the well‐being of the child and the family.”

### 4.2 Discussions

Countless studies across disciplines opine that long‐term illness and emotional well‐being (e.g. happiness) are closely related. Yet, the definition of happiness in children with chronic diseases is being scarcely discussed. The aim of this present study was to define the concept of happiness among children with chronic disease in Ghana, based on their parents’ perspectives. Thus, the hybrid concept analysis approach was used to clarify this concept. Anecdotal evidences have suggested that over the past century, the prevalence of chronic diseases among children and young people has increased relative to infectious diseases. This has left such children with chronic diseases and their families unhappy. Chronic diseases in children have been seen to threaten our traditional notions of happiness (e.g. life satisfaction, felicity, health, well‐being and comfort) (Thorne & Paterson, 1998). Data analyses of the participants’ responses in the fieldwork phase revealed five attributes for the concept of happiness among children with chronic diseases: “Positive quality of life,” “Subjective well‐being,” “Dependence on God,” “Physiological and economic stability” and “Optimism.” Also, from the works of literature reviewed, three attributes were identified: “subjective assessment of meanings/capabilities,” “nurturing social prosperity against the odds” and “hopefulness.” The results of data analysis on the attributes of the concepts from the theoretical and fieldwork studies were closely the same with the fieldwork results, except the emergence of the two‐dimension attribute of “physiological and economic stability” that emanated from the fieldwork phase but did not surface in the theoretical analysis results. It is noteworthy that the first phase and second fieldwork phase attribute both talks about evaluation of a good health, and thus the “Positive quality of life (QL)” and “Subjective well‐being”], respectively. These two attributes are in line with the first theoretical attributes, that is “subjective assessment of capabilities.” From the fieldwork analysis, the parents gave a personal explanation of the current state of their children's health. This emphasizes the basic assertion that personal differences could influence an individual choice of what is important or what they find important. Some people are satisfied with the smallest signs of recovery, while other people expect the best from everything, such as full recovery from the ill health of their children (Lim, 2019). Most parents believe that the “subjective assessment of capabilities” as seen in the theoretical phase means, for example ability to perform some activities of daily living, for example walking and some level of autonomy in self‐care or assisted care despite being sick and their ability to maintain a positive relation to their peers and their family members. In line with this assertion, Felce and Perry (Felce & Perry, 1995) opined that quality of life (QL) is the total well‐being of a person and it involves objective accounts and subjective appraisals of one's physical, worth, personal development and psychological well‐being based on the value placed on such parameters. Yet, in some cases, objective indexes may add‐on, or in the case of persons who are unable to subjectively perceive or serve as a proxy in the assessment of the well‐being of a child (Lim, 2019; Taylor, 2008).

The third field result attribute “Dependences on God” is consistent with the second theoretical attribute “nurturing social prosperity against the odds.” This attribute means that the participants believed that their cultural beliefs and spirituality could be the source of comfort in times of chronic disease crisis, and a foundation for self‐transcendence. They believe that God will intervene for them and will give them a sense of peace. Religion and faith offer an opportunity for spiritual growth and an ability to manage things (Lim, 2019; McCormick & Cushman, 2019). In the Ghanaian context, most mothers with chronically ill children abide by the their traditional moral values of praying and worship to ask for God's help in case of adversity such as caring for a child with chronic disease. This provides the parents with inspiration and also a sense of control and the will power to handle the challenges. The unknown spiritual world cannot be elucidated by pragmatic modern medicine. It is mystical, philosophical and metaphysical aspects of any phenomena, which does not obey conventional interpretations of science and rational thinking. Nevertheless, the spiritual world is real to those having self‐limiting conditions (Eiser & Morse, 2001; McCormick & Cushman, 2019; Peterson et al., 2005).

The fourth two‐dimensional attribute from the fieldwork interview, “Physiological and economic Stability” was not seen in the theoretical literature review analysis. This is in line with the assertion that health consists of at least three wide‐ranging areas: physical, psychological and social functioning that can be altered by the presence of chronic disease. The physical ability includes an altered pattern of rest, sleep and play; altered psychological functions include compulsive hyperactivity, parental and child distress, moral distress, depression and daily burnout; and altered social function includes withdrawal from both quantitative and qualitative aspects of social interactions. The attribute of "Physiological and economic phase" also trails the World Health Organization classification of being healthy in a multidimensional perspective of health such as physical, mental, emotional and social health (Cella & Nowinski, 2002; Eiser & Morse, 2001; Ferrer‐Cascales et al., 2019).

The role of “optimism,” which was the last attribute in the fieldwork phase, also trails the attribute on “hopefulness” seen in the theoretical phase. An optimistic or hopefulness attitude plays a vital role in physical health, and it leads a person to follow a good healthy standard protocol that often leads to a good prognosis. Hopefulness is the overall tendency to believe that experience will lead to a good result. Carver et al. (Carver & Scheier, 2014) explained that to be optimistic is to maintain a generally favourable expectation about the future and a sign of positive expectations. Lots of scholarly works have indicated that optimism is linked with happiness and a higher life expectancy. In a study among patients with coronary bypass, the most optimistic patients assessed before the surgical intervention made more active rehabilitation plans that showed better recovery and had positive and good quality of life than the patients who were not optimistic when the two groups were reassessed after intervention. (McCormick & Cushman, 2019; Scheier et al., 1986). In the final practical definition of happiness, happiness is defined as “the subjective and positive lifelong process of adapting to bio‐psychosocial, physiological, economic and environmental changes caused by the disease trajectory, which affects the well‐being of the child and the family.” Results from the findings show that happiness is a positive psychological concept that can be applied in the nursing field to increase happiness in a chronically ill child and their family.

### 4.3 Usefulness of the concept for research and practice

The hybrid concept analysis method helps to enrich the rigour and trustworthiness of this study. By understanding the meaning of happiness, it will help nurses to be able to assess and know whether the child and the family are able to maintain a balanced point between their individual strength and the challenges faced as a result of the chronic condition so as to ensure a happier state. The understanding of the definition of happiness in children with chronic ill health is “the subjective and positive lifelong process of adapting to bio‐psychosocial, physiological, economic and environmental changes caused by the disease trajectory, which affects the well‐being of the child and the family could also guide health policy decisions so as to bring up an intervention that will help improve their state of being happy.” The findings on the definition and the importance of happiness can be replicated in other paediatric condition and used as a reference point for future research to achieve intensified happiness level in the chronically ill children and their families. This study could also serve as a reference point for the development of useful happiness measurement tools specifically for children with chronic diseases. Overall, this current study highlights clinical opportunities to widen the perspective of health beyond the absence of disease to one where all children, irrespective of their chronic health condition, can have some sense of happiness.

### 4.4 Conclusions

This study aimed to achieve a deeper understanding of the definition of happiness among children with chronic disease, in the Ghanaian chronically sick children and in any context. This study made use of the hybrid method of concept analysis to provide insight into the defining characteristics, attributes and definition of happiness among children with chronic disease bases on their parents’ perspectives. Happiness as a concept is multidisciplinary, and both empirical researchers and laypersons use happiness as a proxy for describing the state of well‐being. Children with stable emotions, that is happiness state, have shown a good outcome/prognosis for their chronic health condition. Happiness among children with chronic diseases is defined practically after empirical and fieldwork analysis in this paper as the “subjective and positive lifelong process of adapting to bio‐psychosocial, physiological, economic and environmental changes caused by the disease trajectory, which affects the complete well‐being of the child and the family.” It is believed that this definition will help nurses in assessing the child and the family with chronic disease to improve the child's and family's emotional state of happiness.

### 4.5 Limitations of the study

The fieldwork phase of this study was mainly done using qualitative design method, which has a limitation of small sample size. This limits generalization of the meaning of the concept of happiness to other cultural settings with children population with chronic disease.

### 4.6 Recommendations

It is recommended that another study is conducted with the perspectives of children alongside their parents and different settings and possibly assessment of happiness in other childhood conditions, to gain more understanding of the concept. It is also recommended that evaluation screening tools be explicitly developed to measure happiness among children with the chronic disease so as to serve as a tool for assessing happiness in children with chronic illness to plan appropriate prescribed management for the child and the family.

## CONFLICT OF INTEREST

The authors declare no conflict of interest.

## AUTHOR CONTRIBUTIONS

R.N.N, M.A.C and A.S.H: Conceptualization. R.N.N., M.A.C and A.S.H: Methodology. R.N.N., M.A.C., S.P. and A.S.H.: Validation. R.N.N., M.A.C and A.S.H: Formal analysis. R.N.N., M.A.C and A.S.H: Investigation. R.N.N: Resources. R.N.N. and M.A.C: Data curation. R.N.N., M.A.C and A.S.H: Writing—original draft preparation. R.N.N., M.A.C, S.P and A.S.H: Writing and editing. R.N.N.: Visualization. M.A.C., S.P. and A.S.H.: Supervision. R.N.N: Project administration. All authors have read and agreed to the published version of the manuscript.
